# Krüppel-like factors in mitochondrial quality control

**DOI:** 10.3389/fphys.2025.1554877

**Published:** 2025-04-08

**Authors:** M. Y. Zhang, H. Zhang, Y. M. Yao, D. W. Yang

**Affiliations:** ^1^ Department of Nephrology, Tianjin Hospital of Tianjin University, Tianjin, China; ^2^ Translational Medicine Research Center, Medical Innovation Research Division and the Fourth Medical Center of Chinese PLA General Hospital, Beijing, China

**Keywords:** Krüppel-like factors, mitochondrial quality control, mitochondrial biogenesis, mitochondrial fusion/fission, mitophagy, mitochondrial unfolded protein response

## Abstract

Krüppel-like factors (KLFs) are a group of transcription factors characterized by conserved zinc finger domains in the C-terminus, which are critically involved in basic cellular processes, including growth, differentiation, apoptosis, and angiogenesis, and play important roles in many pathophysiological responses. Mitochondrial homeostasis relies on a coordinated mitochondrial quality control system, which maintains the number and morphological stability and coordinates mitochondrial physiological functions through renewal and self-clearance. In this paper, we review the current advances of KLFs in mitochondrial quality control (MQC), including the potential roles and regulatory mechanisms in mitochondrial biogenesis, mitochondrial fusion/fission, mitophagy and mitochondrial unfolded protein response. We also introduce the specific pharmacological modulation of KLFs, expecting to transforming basic research achievements and providing the possibility of targeted therapy for KLFs.

## 1 Introduction

Krüppel-like factors (KLFs) are a group of transcription factors characterized by conserved zinc finger domains in the C-terminus. Up to now, 18 mammalian KLFs have been identified, which are broadly involved in fundamental physiological processes, and play important roles in many pathophysiological responses. Although it has been gradually revealed the role of the KLF family in the mitochondrial homeostasis under physiological and pathological conditions, there is still a lack of review articles to sort out and summarize the relevant advances. To fill this gap, we review the current research findings on the KLF family in MQC, including their roles and regulatory mechanisms in mitochondrial biogenesis, mitochondrial fusion/fission (dynamics), and mitophagy. It is expected to provide evidence for repairing mitochondrial dysfunction by regulating KLFs.

## 2 KLF family

**FIGURE 1:**
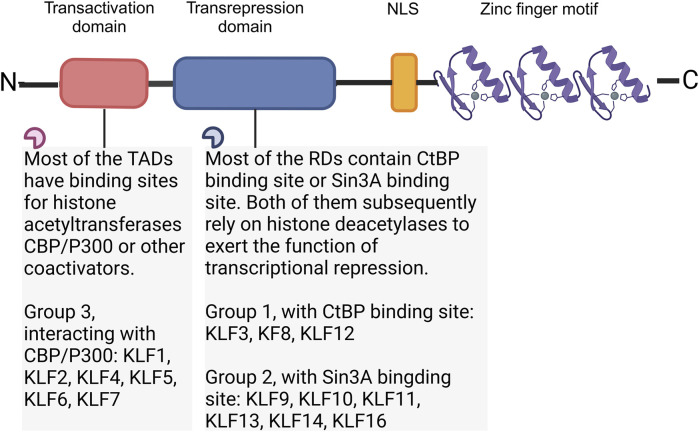
Structure of KLF family. According to the interaction with transcriptional co-activators and/or co-repressors, KLFs can be divided into three groups. Group 1 includes KLFs that contain the CtBP binding site, group 2 includes KLFs that contain the Sin3A interaction domain, and group 3 includes KLFs which are capable of interacting with acetyltransferases and generally recognized as transcriptional activators. Figure was created in BioRender. Zhang, M. (2025) https://BioRender.com/v55j346. NLS: Nuclear localization signal; CtBP: C-terminal binding protein; Sin3A: SIN3 transcription regulator family member A; CBP: CREB-binding protein.

“Krüppel” is first described as “cripple”, and it is crucial to body segmentation in *Drosophila*, which might results in embryonic lethality if mutations at very early stage of embryogenesis (Kopp, 2015). In mammalian, orthologs of *Drosophila* Krüppel refer to KLFs, 18 mammalian KLFs have been identified up to now. KLF1 to 17 are numbered and named in the order of discovery, KLF18 exists in most of the genomic sequenced mammals, but no protein expression has been reported. The chromosomal location of KLF18 gene is adjacent to KLF17, makes it probably a product of duplication of KLF17 (Pei and Grishin, 2013). Except for the erythroid-specific KLF (KLF1), which is mainly presented in erythrocytes and megakaryocytes (Perkins et al., 2016), most of the other KLFs are widely expressed in tissue cells. KLFs exhibit tissue-specificity, participating in fundamental physiological processes, inflammatory response, oxidative stress, apoptosis and carcinogenesis (Liang et al., 2018). The carboxyl terminus of KLFs contains three C2H2-type zinc finger domains, which directly bind to GC-rich genic sites, mediating KLFs in the modulation of target gene at promoters, enhancers, and locus control regions. The amino terminus contains the transactivation domains and/or transrepression domains and various protein interaction domains (Figure 1). These domains allow KLFs to bind with molecular chaperones that modify DNA expression, including transcription factors, coactivators, and coinhibitors, like CREB-binding protein (CBP), SIN3 transcription regulator family member A (Sin3A), and C-terminal binding protein (CtBP) as well as chromatin modifying enzymes including p300 and histone deacetylases (Mas et al., 2011; Fan et al., 2017; Oishi and Manabe, 2018) (Table 1).

**TABLE 1:** Structural characteristics of amino-terminus in different KLFs.

KLFs	Characteristic description
KLF1	KLF1 has two N-terminal transactivation domains (TADs). However, the post-translational modification of KLF1 can mediate its binding to protein inhibitor of activated STAT (PIAS) family members, p300/CREB and SWI/SNF-related chromatin remodeling complexes to exert transcriptional repression effect^(a)^
KLF2	The N-terminus of KLF2 has an adjacent TAD and a repression domain (RD). The latter possesses the ability to bind specific ubiquitin ligases mediating proteolytic degradation^(b)^
KLF3	KLF3 only has a RD, and its interaction with CtBP is the key to repressing transcription. In addition, the SUMOylation of KLF3 and its binding to the protein Four and A Half LIM Domains 3 (FHL3) are also crucial for KLF3-mediated transcriptional repression of target genes^(c)^
KLF4	KLF4 possesses adjacent TAD and RD and two nuclear localization signals (NLS)^(d)^
KLF5	KLF5 has a proline-rich TAD, which recruits the E3 ubiquitin ligase WWP1 and SCFFBW7. KLF5 is regulated by ubiquitination, phosphorylation, and SUMOylation^(e)^
KLF6	The protein structure of KLF6 only contains a TAD, which is responsible for recruiting cofactors, such as KLF4, p53, HIF1α, and histone deacetylase 3 (HDAC3), and it activates or inhibits the transcription process in a context-dependent manner ^(f)^
KLF7	KLF7 processes acidic domain and hydrophobic serine-rich domain, and it functions as transcriptional activator predominantly. There is an evolutionarily conserved leucine zipper between the 59th to 119th amino acid residues of KLF7, which interacts with the cofactor F-box protein 38 (FBXO38). FBXO38 not only enhances the transcriptional activation of KLF7, but also regulates the subcellular localization in the nucleus and cytoplasm^(g)^
KLF8	The regulatory region of KLF8 contains both an RD that binds to CtBP and a TAD that can recruit transcriptional co-activators P300 and PCAF histone acetyltransferases. The acetylation and SUMOylating sites of KLF8 have also been identified^(h)^
KLF9	There is a conserved α-helical repression motif in the 3rd-21st amino acids of KLF9, which interacts with the inhibitor Sin3A. It has been reported that KLF9 can recruit coactivator cAMP-response element binding protein-binding protein with the help of some transcription factors. Nucleosome histone acetylation in the gene regulatory region plays a positive regulatory role^(i)^
KLF10	KLF10 contains multiple proline-rich Src homology-3 (SH3) binding domains which bind Sp1 for transcriptional regulation. In addition, there are three unique RDs with interaction sites with Sin3A throughout the protein structure^(j)^
KLF11	There exist three RDs in the N-terminal region of KLF11. They can be tethered to the DNA and mediate repression. In which, the R1 domain has been shown to be essential for the interaction with the co-repressor Sin3A^(k)^
KLF12	KLF12 interacts with CtBP through the amino-terminal PVDLS sequence (Pro-Xaa-Asp-Leu-Ser), thereby inhibiting the transcription of target genes^(l)^
KLF13	In KLF13, a TAD, a RD, and two NLSs have been identified. Its RD can interact with Sin3A to exert its transcriptional repression activity. At the same time, the zinc finger domain has also been shown to mediate interaction with coactivators such as CBP/p300 ^(m)^
KLF14	KLF14 exerts its transcriptional repression activity by interacting with Sin3A^(n)^
KLF15	KLF15 comprises serine-rich or proline-rich sequences which are considered as repression motifs. Additionally, there exists a glutamic acid cluster in amino acid residues 142 to 150 of KLF15, which may play an inhibitory role in transcriptional regulation^(o)^
KLF16	KLF16 simultaneously possesses a TAD that couples with histone acetyltransferase-signaling pathway, as well as a RD with Sin3A interacting domain^(p)^
KLF17	A detailed description is still lacking
KLF18	No protein expression data of KLF18 has been reported. The chromosomal location of KLF18 gene is adjacent to KLF17, makes it probably a product of duplication of KLF17^(q)^

^a^ Caria CA, Faà V, Ristaldi. Krüppel-like factor 1: a pivotal gene regulator in erythropoiesis. Cells. (2022) 11:3069. doi:10.3390/cells11193069.

^b^ Turpaev KT., Transcription factor KLF2 and its role in the regulation of inflammatory processes. Biochemistry (Mosc). (2020) 85:54-67. doi:10.1134/s0006297920010058.

^c^ Hu LJ., Research progress on the structure and physiological function of KLF3. Chin J Immunol. (2013) 29:989-993. doi:10.3969/j.issn.1000-484X.2013.09.022.

^d^ Ghaleb AM, Yang VW., Krüppel-like factor 4 (KLF4): What we currently know. Gene. (2017) 611:27-37. doi:10.1016/j.gene.2017.02.025.

^e^ Luo Y, Chen C. The roles and regulation of the KLF5 transcription factor in cancers. Cancer Sci. (2021) 112:2097-2117. doi:10.1111/cas.14910.

^f^ Syafruddin et al., 2020 f. Syafruddin SE, Mohtar MA, Wan Mohamad Nazarie WF, Low TY., Two sides of the same coin: the roles of KLF6 in physiology and pathophysiology. Biomolecules. (2020) 10:1378. doi:10.3390/biom10101378.

^g^ Chen YC, Wei H, Zhang ZW., Research progress of Krüppel-like factor 7. Sheng Li Xue Bao. (2016) 68:809-815.

^h^ Lahiri SK, Zhao J. Krüppel-like factor 8 emerges as an important regulator of cancer. Am J Transl Res. (2012) 4:357-363.

^i^ Kang L, Lai MD. BTEB/KLF9 and its transcriptional regulation. Hereditas. (2007) 29:515-522. doi:10.3321/j.issn:0253-9772.2007.05.002.

^j^ Subramaniam M, Hawse JR, Rajamannan NM, Ingle JN, Spelsberg TC., Functional role of KLF10 in multiple disease processes. Biofactors. (2010) 36:8-18. doi:10.1002/biof.67.

^k^ Lin L, Mahner S, Jeschke U, Hester A. The distinct roles of transcriptional factor KLF11 in normal cell growth regulation and cancer as a mediator of TGF-β, signaling pathway. Int J Mol Sci. (2020) 21:2928. doi:10.3390/ijms21082928.

^l^ Ding Y, Ding L, Cheng ZY, Zhou HJ., Research progress on the function of zinc finger protein KLF12. Journal of Southeast University (Medical Science Edition). (2017) 36:1036-1039. doi:10.3969/j.issn.1671-6264.2017.06.035.

^m^ Song A, Patel A, Thamatrakoln K, Liu C, Feng D, Clayberger C, et al. Functional domains and DNA-binding sequences of RFLAT-1/KLF13, a Krüppel-like transcription factor of activated T lymphocytes. J Biol Chem. (2002) 277:30055-30065. doi:10.1074/jbc.M204278200.

^n^ Chen X, Shi W, Zhang H. The role of KLF14 in multiple disease processes. Biofactors. (2020) 46:276-282. doi:10.1002/biof.1612.

^o^ Chen H, Li LL, Du Y. Krüppel-like factor 15 in liver diseases: Insights into metabolic reprogramming. Front Pharmacol. (2023) 14:1115226. doi:10.3389/fphar.2023.1115226.

^p^ Daftary GS, Lomberk GA, Buttar NS, Allen TW, Grzenda A, Zhang J, et al. Detailed structural-functional analysis of the Krüppel-like factor 16 (KLF16) transcription factor reveals novel mechanisms for silencing Sp/KLF, sites involved in metabolism and endocrinology. J Biol Chem. (2012) 287:7010-7025. doi:10.1074/jbc.M111.266007.

^q^ Pei J, Grishin NV. A new family of predicted Krüppel-like factor genes and pseudogenes in placental mammals. PLoS One. (2013) 8:e81109. doi:10.1371/journal.pone.0081109.

## 3 Mitochondrial quality control (MQC)

**FIGURE 2:**
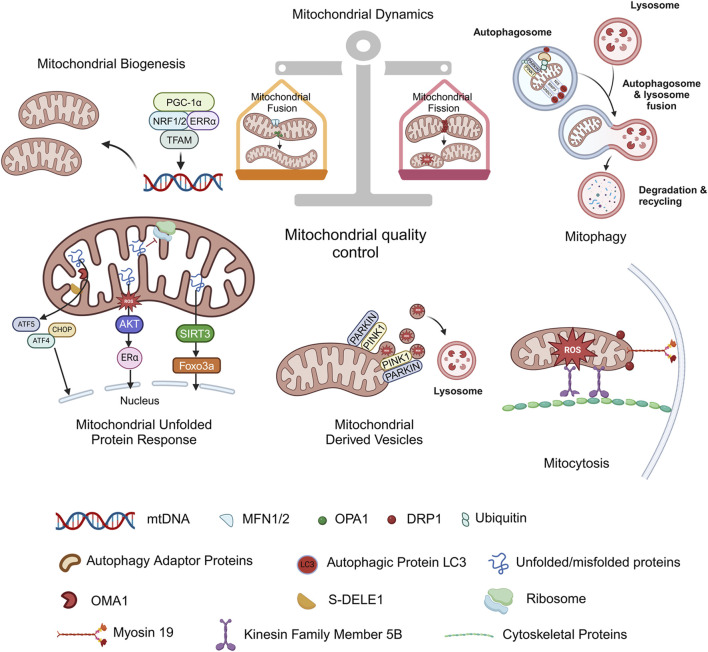
Mitochondrial quality control. Mitochondrial homeostasis appears to be dependent on a coordinated mitochondrial quality control system, which mainly involves mitochondrial biogenesis, mitochondrial fusion/fission, and mitophagy at the organelle level. Mitochondrial unfolded protein response (UPRmt), Mitochondrial-derived vesicles (MDVs) and mitocytosis are discovered stress-induced mitochondrial regulation in recent years, and they are also considered as new members of the mitochondrial quality control system. Figure was created in BioRender. Zhang, M. (2025) https://BioRender.com/f27i573. mtDNA: mitochondrial deoxyribonucleic acid; PGC-1α: proliferator-activated receptor-γ coactivator-1α; NRF1/2: nuclear respiratory factors 1/2; ERRα: estrogen-related receptor α; TFAM: mitochondrial transcription factor A; PINK1: PTEN-induced putative kinase 1; PARKIN: parkin RBR E3 ubiquitin protein ligase; NIX: NIP3-like protein X; BNIP3: BCL2/adenovirus E1B 19 kDa protein interacting protein 3; FUNDC1: FUN14 domain-containing 1; MFN1/2: mitofusin 1/2; OPA1: optic atrophy protein 1; DRP1: dynamin related protein 1; S-DELE1: short fragments of DAP3 binding cell death enhancer 1; ATF4: activating transcription factor 4; ATF5: activating transcription factor 5; CHOP: C/EBP homologous protein; AKT: protein kinase B; Erα: estrogen receptor alpha; SIRT3: sirtuin 3; FOXO3a: forkhead box O3a.

Mitochondria exist in almost all eukaryotes. As the site of intracellular oxidative phosphorylation, mitochondria play pivotal roles in cell survival and death by providing energy. Mitochondrial homeostasis appears to be dependent on a coordinated and sophisticated mitochondrial quality control system, which mainly involves processes such as mitochondrial biogenesis, mitochondrial fusion/fission and mitophagy at the organelle level (Figure 2).

### 3.1 Mitochondrial biogenesis

Mitochondrial biogenesis is the major source of new mitochondria. As the basis for ensuring cell energy supply, it has a momentous part in cell proliferation, division, and pathological conditions such as starvation as well as oxidative stress. Mitochondrial biogenesis is a kind of self-renewal, through which new mitochondria are generated from the existing ones (Popov, 2020). Mitochondrial biogenesis is largely dependent on the coordination between the mitochondrial and the nuclear genomes, and proliferator-activated receptor-γ coactivator-(PGC-)1α is considered a principal controller. By activating various transcription factors, including nuclear respiratory factors 1 (NRF1) and NRF2, and estrogen-related receptor α (ERRα), PGC-1α upregulates the expression of mitochondrial transcription factor A (TFAM) to drive mitochondrial deoxyribonucleic acid (mtDNA) replication and transcription, thereby promoting mitochondrial biosynthesis (Tian et al., 2022; Chodari et al., 2021). The elevated mtDNA copy numbers, mtDNA: nuclear DNA ratio, and the expression of mitochondrial genes are often chosen as reliable markers of mitochondrial biogenesis (Andres et al., 2017), enhanced expression of PGC-1α, NRF1/2, and TFAM may also serve as indirect evidences.

### 3.2 Mitochondrial fusion/fission

Mitochondrial fusion/fission refers to mitochondria undergoing dynamic mechanical alterations of the architecture. Mitochondrial fusion can form an interconnected network structure and promote the redistribution of proteins and mitochondrial DNA, which is propitious to mitochondrial repair and adaptation to higher metabolic requirements. Mitochondrial fission makes mitochondria tend to be fragmented. On the one hand, it is conducive to mitochondrial transport to high energy-consuming regions and mitosis; on the other hand, it separates dysfunctional or damaged mitochondrial components for subsequent clearance (Romanello, 2020). Mitochondria undergo continuous changes in mass and morphology through fusion and fission. The subtle balance of the dynamic alterations is crucial to cell survival and optimization of function. It has been demonstrated that mitochondrial fusion is coordinated by outer mitochondrial membrane (OMM) located fusion protein, mitofusin 1/2 (MFN1/2), and optic atrophy protein 1 (OPA1), which bind to cardiolipin promoting inner mitochondrial membrane (IMM) intermingling (Adebayo et al., 2021). One factor in mitochondrial fission is the cessation of fusion, which decreases the expression of mitofusins after cells are stressed, in turn inhibiting fusion and augmenting mitochondrial fission. Another important factor is the formation of helices around OMM by dynamin related protein 1 (DRP1) in the cytoplasm or endoplasmic reticulum and their interactions with OMM-located adaptor proteins, for example, fission protein 1 (FIS1), mitochondria fission factor (MFF), and mitochondrial dynamics proteins of 49 and 51 (MID49/51). DRP1 binds to adaptor proteins and exerts mechanical forces to complete mitochondrial fission (Wang and Zhou, 2020).

### 3.3 Mitophagy

Mitophagy is a selective degradation mechanism for dysfunctional mitochondria in cells (Onishi et al., 2021). It reduces mitochondrial reactive oxygen species (ROS) accumulation and restores energy supply by removing damaged mitochondria. Mitophagy is one of the highly conserved organelle‐specific autophagy pathways, which is crucial in early embryonic development, cell differentiation, and apoptosis by removing damaged or non-essential mitochondria from cells (Onishi et al., 2021). The mitophagy mentioned below mainly refers to macromitophagy, characterized by the formation of autophagosome with a bilayer membrane, which eventually shifts target mitochondria to lysosomes for degradation. According to the targeting signals and subsequent pathways, mitophagy can be classified into three categories: ubiquitin-dependent mitophagy, receptor based mitophagy, and lipid based mitophagy. PTEN-induced putative kinase 1 (PINK1)-PARKIN signaling is the most widely studied mitophagy pathway among all others. With the deepening of discovery, several signaling pathways that are ubiquitin dependent, while PINK1 and/or PARKIN independent have been gradually reported, such as glycoprotein 78 (GRP78) mitophagy, mitochondrial E3 ubiquitin ligase 1 (MUL1) based mitophagy, and p62/SQSTM1 based mitophagy. In receptor mediated mitophagy, is has been identified several types of mitophagy-related receptors in mammals, including BCL2/adenovirus E1B 19 kDa protein interacting protein 3 (BNIP3), NIP3-like protein X (NIX)/BNIP3L, FUN14 domain-containing 1 (FUNDC1), BCL2 like 13 (BCL2L13), FKBP prolyl isomerase 8 (FKBP8), prohibitin 2 (PHB2), and BECLIN1-regulated autophagy 1 (AMBRA1), etc. In the last part, certain lipids that directly combine with microtubule-associated protein light chain 3 (LC3) as mitophagy receptors mainly include cardiolipin and ceramides (Choubey and Zeb, 2021). Defects in mitophagy play a role in occurrence and progress of various pathological conditions, and appropriate modulation is a potential therapeutic choice for cell death and tissue damage.

### 3.4 What’s new in MQC

The mitochondrial unfolded protein response (UPRmt) serves as a pivotal mechanism in MQC. It is activated by the aberrant accumulation of unfolded/misfolded proteins within the mitochondrial matrix, initiating retrograde signaling to the nucleus to upregulate the expression of proteases and molecular chaperones. This process mitigates proteostatic stress and ensures mitochondrial functional homeostasis. To date, multiple UPRmt axes have been identified, including the canonical UPRmt axis, translational axis, SIRT3 axis, and UPR^IMS^/ERα axis (Torres et al., 2024).

Mitochondrial-derived vesicles (MDVs) are single-layer membrane structures from the mitochondrial outer membrane or double-layer membrane structures including inner and outer membranes as well as matrix contents, with a diameter of only 70–150 nm. MDVs function at the very early stages of stress by selectively carrying oxidation products to lysosomes for degradation, and are considered to be the first-round defense mechanism for MQC (Picca et al., 2020; Guan et al., 2021). The mitochondria spheroid formation is also a novel pathway for MQC induced by severe stress which may undergo the fusion with a lysosome during its formation (Yin and Ding, 2013).

Mitocytosis, first reported in 2021, is described as a regulatory mechanism underlying migrating cells eliminate damaged mitochondria into the surrounding environment. It occurs in basal and mild stress conditions, mediated by kinesin family member 5B (KIF5B), myosin 19 (Myo19), and DRP1, mitochondria are pulled to the periphery of the cell and enter the migrasomes, where they are ejected into the extracellular environment as cells migrate, becoming a new member of the quality control system (Jiao et al., 2021). In neurons, mitochondrial Rho (Miro) forms a complex with dynein or kinesin motor proteins, which in turn interacts with Milton and TRACK adaptors to transport mitochondria anterogradely or retrogradely to meet the energy demands of different subcellular regions. This transport mechanism is also regarded as a part of the generalized mitochondrial quality control system (Loss and Stephenson, 2017; Leites and Morais, 2018).

Taken together, MQC can maintain the number and morphological stability and coordinates mitochondrial physiological functions through renewal and self-clearance. Once the mitochondrial quality control system is disordered, mitochondrial dysfunction will occur rapidly, damage energy supply, affect cell metabolism, and may eventually result in cell death and tissue injury via potential mechanisms including mitochondrial permeability transition, calcium overload, excessive accumulation of ROS, lipid peroxidation, and massive release of pro-apoptotic factors.

## 4 KLFs in mitochondrial quality control

### 4.1 KLFs in mitochondrial biogenesis

**FIGURE 3:**
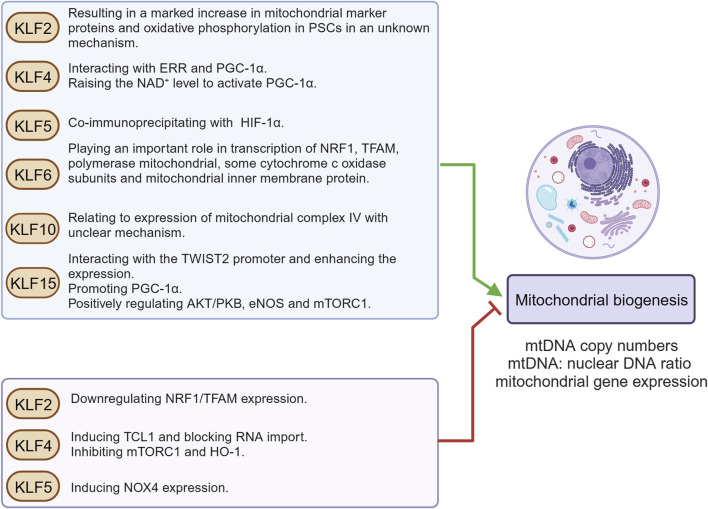
KLFs in mitochondrial biogenesis. Mitochondrial biogenesis is a self-renewal route, by which new mitochondria are generated from the ones already existing. This figure summarizes KLFs that may be involved in the modulation of mitochondrial biogenesis and their regulatory mechanisms. Figure was created in BioRender. Zhang, M. (2025) https://BioRender.com/f38g035. mtDNA: mitochondrial deoxyribonucleic acid; NRF1: nuclear respiratory factors 1; TFAM: mitochondrial transcription factor A; PGC-1α: proliferator-activated receptor-γ coactivator-1α; ERR: estrogen-related receptor; PSCs: pluripotent stem cells; NAD^+^: nicotinamide adenine dinucleotide; TCL1: T-cell leukemia/lymphoma 1 gene; mTORC1: mechanistic/mammalian target of rapamycin complex 1; HO-1: heme oxygenase 1; NOX4: NADPH oxidase 4; HIF-1α: hypoxia-inducible factor-1α; TWIST2: Twist-related protein 2; AKT/PKB: protein kinase B; eNOS: endothelial nitric oxide synthase.

Mitochondrial biogenesis serves as the primary source of new mitochondria and largely depends on the coordination between mitochondrial and nuclear genomes. Multiple KLFs play regulatory roles in mitochondrial biogenesis, exhibiting high cell specificity and context dependence (Figure 3). KLF2 is abundantly expressed in lung tissue, vascular endothelial cells, and myeloid cells and initially identified as a regulator of lung development (Wani et al., 1999). Increasing evidence has revealed the multiple effects of KLF2, making it gradually become a potential therapeutic target for multiple diseases (Yan et al., 2020; Lopez-Ramirez et al., 2021). Niemann-Pick type C (NPC) and acid sphingomyelinase (ASM) deficiencies are two kinds of lysosomal storage diseases caused by mutations in lysosomal protein-coding genes. Yambire et al. found that upregulation of KLF2 expression in cells and tissues from both of these diseases and proposed that KLF2 inhibited mitochondrial biogenesis by down-regulating NRF1/TFAM expression (Yambire et al., 2019). As we known, KLF2 is induced via the mitogen-activated protein kinase kinase kinase 2/3 (MEKK2/3)-mitogen-activated protein kinase kinase 5 (MEK5)-extracellular-signal-regulated kinase 5 (ERK5) signaling pathway in vascular endothelial cells under blood flow-mediated sustained laminar shear stress (Akmeriç and Gerhardt, 2022). KLF2 correlate with reduced mitochondrial content, respiratory function, and endothelial cell metabolic activity (Doddaballapur et al., 2015), nevertheless, whether KLF2 directly regulates mitochondrial biogenesis in vascular endothelial cells is still unclear. On the contrary, studies have indicated that KLF2 plays a key role in maintaining the ground state pluripotency of mouse embryonic stem cells and can reset the self-renewal requirements of human pluripotent stem cells (PSCs). Resettlement of human PSCs by activation of NANOG and KLF2 results in a marked elevation in mitochondrial marker proteins and an increase in oxidative phosphorylation (Yeo et al., 2014; Takashima et al., 2014). Therefore, we believe that KLF2 is indeed involved in the modulation of mitochondrial biogenesis, but the differences and mechanisms in somatic cells and PSCs need to be further elucidated (Figure 3).

KLF4 was first reported as an epithelial-specific transcription factor in 1996 (Shields et al., 1996). In recent years, KLF4 has gradually become a research hotspot, which has been confirmed to be participation in regulating cell proliferation and differentiation, promoting wound healing, bone development, spermatogenesis, and maintaining genetic stability (Ghaleb and Yang, 2017). KLF4 also participates in the inflammatory response by regulating macrophage polarization (Li et al., 2018; Kapoor et al., 2015), release of inflammatory mediators (Liu et al., 2008; Liu et al., 2007; Liu et al., 2012; Czakai et al., 2016), phenotypic switching of vascular smooth muscle cells (VSMCs) (Li et al., 2010; Yang et al., 2021), oxidative stress injury (Cao et al., 2021), apoptosis (Tong et al., 2020), and autophagy (Gan et al., 2017; Zhao et al., 2019). In myocardial tissues, KLF4 is essential for mitochondrial biogenesis and physiological respiratory function (Ding et al., 2021). Mechanistically, KLF4 interplays with ERR and PGC-1α, and then the three-protein complex binds to the DNA promoter region that encodes mitochondrial proteins to regulate transcription and enhance mitochondrial biogenesis (Liao et al., 2015). Another study revealed that after specific knockout of KLF4 in cardiomyocytes, the expression of SIRT3 and SIRT5 was decreased together with reduction in nicotinamide adenine dinucleotide (NAD^+^) (Zhang et al., 2017). NAD^+^ can activate PGC1α (Bai et al., 2011), and it indirectly proves that KLF4 plays a positive regulatory role in mitochondrial biogenesis. Likely, KLF4 promotes mitochondrial self-renewal and improves oxidative stress in renal vascular endothelial cells. Overexpression of KLF4 significantly reduces renal triglyceride deposition, protects against obesity-related nephropathy, and can be used as a prognostic marker to guide clinical evaluation (Jin et al., 2020a). On the contrary, overexpression of KLF4 inhibits mitochondrial biogenesis in retinal ganglion cells, and a decrease in mitochondrial complex size has been noted (Steketee et al., 2012). In adipose conversion, KLF4 expression is upregulated during the conversion to white adipose phase, accompanied by a significant decrease in mitochondrial abundance and low expression of genes responsible for oxidative phosphorylation (Basse et al., 2015). Additionally, Nishimura et al. reported that KLF4 induced T-cell leukemia/lymphoma 1 gene (TCL1) transcription by directly binding to its enhancer and promoter regions during somatic reprogramming. TCL1 antagonizes mitochondrial polynucleotide phosphorylase, blocks RNA transport into mitochondria, finally inhibits mitochondrial biogenesis (Nishimura et al., 2017). Meanwhile, KLF4 and c-Myc inhibit the activation of mechanistic/mammalian target of rapamycin complex 1 (mTORC1), which negatively regulates mitochondrial biogenesis during reprogramming (Wu et al., 2015). Indirect evidence suggests that KLF4 may eventually downregulate heme oxygenase 1 (HO-1) expression by inhibiting ELK-3 expression in macrophages (Tsoyi et al., 2015), and HO-1 is closely associated with the regulation of mitochondrial homeostasis (Shi et al., 2019). The reasons for these contradictory conclusions are diverse. Setting aside the differences in experimental conditions, first of all, based on the protein structure of KLF4, within its amino terminus, KLF4 simultaneously possesses a transactivation domain and a repression domain, together of which determine the specificity of KLF4’s transcriptional regulating activity by interacting with other factors. Secondly, as a transcriptional regulator, KLF4 has different target genes in various cells. At the same time, KLF4 itself has multiple phosphorylation, acetylation, ubiquitination, and SUMOylation sites (Ghaleb and Yang, 2017). The specific post-translational modifications under different tissues or pathophysiological states may all lead to transcriptional differences of downstream molecules. In addition, there are also differences in mitochondrial abundance, metabolic environment, ion concentration, pH, etc. in different tissues. During somatic cell reprogramming, the signaling pathways involved are even more complex. KLF4 may participate in multiple pathways and regulate a variety of downstream genes. Similarly, during somatic cell reprogramming or under certain other special pathophysiological conditions, there may also be specific microRNAs (miRNAs) or long non-coding RNAs (lncRNAs) that can inhibit or enhance the functions of target molecules, or abnormal methylation which may directly affect the transcriptional initiation of genes downstream of KLF4. These results indicate that regulation of KLF4 in mitochondrial biogenesis is highly cell specific and context dependent.

KLF5 was first cloned in 1993 and participated in regulation of multiple cellular processes, including lung development, adipogenesis, and carcinogenesis (Dong, 2006). Recently, accumulating evidence demonstrates that the increased expression of KLF5 is positively correlated with diabetic myocardium injury, atherosclerotic lesions and end-stage heart failure. Inhibition of KLF5 expression has become an emerging treatment for cardiovascular diseases (Palioura et al., 2022). Via directly binding to the promoter, KLF5 induce NADPH oxidase (NOX)4 expression in cardiomyocytes. KLF5/NOX4 not only aggravates oxidative stress injury, but also damages mitochondrial membrane potential, inhibits respiratory function, and reduces mitochondrial abundance (lower mitochondrial DNA: nuclear DNA ratio and weakened mitochondrial proteins) (Kyriazis et al., 2021). Indeed, KLF5 was noticed to co-immunoprecipitated with hypoxia-inducible factor-1α (HIF-1α) in non-small cell lung cancer cells, and KLF5 inhibition downregulated several downstream genes of HIF-1α (Li et al., 2014), while the regulation of HIF-1α on mitochondrial biogenesis varied with cell type and pathophysiological environment.

KLF6 is originally defined as a key transcription factor participated in the regulation of pregnancy-specific glycoprotein gene expression (Koritschoner et al., 1997). Subsequently, KLF6 is found to play a role in multiple physiological processes, such as cellular differentiation and proliferation, immune and inflammatory responses, tissue injury, and wound healing. In addition, genetic alterations and/or the aberrant expression of KLF6 have been implicated in the pathogenesis of many cancer types and several inflammatory associated diseases (Syafruddin et al., 2020). Mallipattu et al. observed a significant decrease in KLF6 expression in renal podocytes of HIV-associated nephropathy (HIVAN) and focal segmental glomerulosclerosis (FSGS) patients. The study also revealed that upon doxorubicin stimulation, the transcription level of NRF1, TFAM, polymerase mitochondrial, some cytochrome c oxidase subunits, and mitochondrial inner membrane protein (Mpv17) in podocytes of KLF6 knockout mice were obviously reduced (Mallipattu et al., 2015), suggesting that KLF6 might be critical for mitochondrial biogenesis in podocytes. In contrast, KLF6 reduces mitochondrial complex size and mitochondrial density in retinal ganglion cell (Steketee et al., 2012), the possible mechanism of which needs further investigation.

KLF10, originally named transforming growth factor inducible early gene 1 (TIEG1) has a widespread expression in mammals and participates in various cell biological processes, coordination of circadian rhythms and metabolic homeostasis, and is associated with obesity, insulin resistance, osteoporosis, cardiac hypertrophy, angiogenesis, and different types of tumors (Memon and Lee, 2018). Recently, it was reported that KLF10 was an emerging regulator of immunocyte function. KLF10 deficiency in CD4^+^-T cells significantly impaired the differentiation of regulatory T cells (Tregs) and was accompanied by marked inhibition of mitochondrial respiration with loss of mitochondrial mass (Wara et al., 2020). Similarly, in soleus muscle, the regulation of KLF10 on mitochondrial mass and function was reproduced, but it was noted that KLF10 knockout only significantly reduced expression of mitochondrial complex IV (cytochrome c oxidase), but no significant differences of PGC1α were observed, implicating that the impact and its mechanism with regard to KLF10 on mitochondrial biogenesis remains unclear (Kammoun et al., 2020).

KLF15 is considered to be an important regulator and effector of sucrose, lipid, and amino acid metabolism in organs, highly expressed in hypermetabolic tissues (such as liver, kidney, heart, and skeletal muscle), and closely related to adipogenesis and insulin sensitivity of adipocytes (Hsieh et al., 2019). Zhou et al. found that KLF15 directly interacted with the Twist-related protein 2 (TWIST2) promoter and enhanced its expression. Meanwhile, TWIST2 improved mitochondrial quantity and function in hepatocytes in a fibroblast growth factor 21 (FGF21)-dependent manner, not only promoting mitochondrial biogenesis and enhancing ATP production, but also improving antioxidant capacity and reducing intracellular ROS level (Zhou et al., 2019). Moreover, KLF15 expression was increased in hepatocytes treated with glucagon, and high binding of PGC-1α to KLF15 RNA was confirmed by enhanced ultraviolet cross-linking and immunoprecipitation followed by sequencing (eCLIP-seq) (Tavares et al., 2020). Also, KLF15 seems to regulate mitochondrial biogenesis in heart, which may protect against doxorubicin (DOX)-induced cardiac toxicity by augmenting mitochondrial biogenesis (PGC-1α and COX-IV expression) and improving respiratory function. It was shown by Tedesco et al. that KLF15 was significantly reduced by DOX treatment in cardiomyocytes. Downregulation of KLF15 negatively modulated protein kinase B (AKT/PKB), endothelial nitric oxide synthase (eNOS), and mTORC1 successively, thus inhibiting mitochondrial biogenesis and aggravating oxidative stress (Tedesco et al., 2020).

### 4.2 KLFs in mitochondrial fusion/fission

**FIGURE 4:**
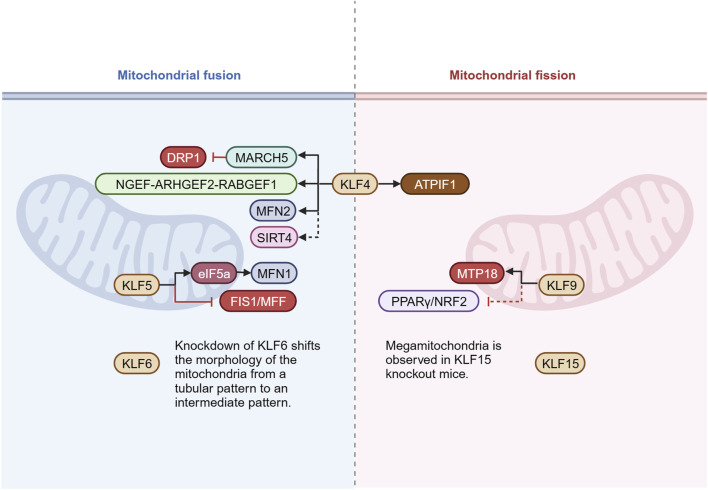
KLFs in mitochondrial fusion/fission. Mitochondria undergo continuous alterations in mass and morphology through fusion and fission. The delicate balance between fusion and fission is essential for cell survival and optimization of function. This figure presents the known KLFs and their target molecules involved in the regulation of mitochondrial fusion/fission. Figure was created in BioRender. Zhang, M. (2025) https://BioRender.com/a49o680. DRP1: dynamin related protein 1; March5: membrane-associated ring finger (C3HC4) 5; NGEF: neuronal guanine nucleotide exchange factor; ARHGEF2: Rho/Rac guanine nucleotide exchange factor 2; RABGEF1: RAB guanine nucleotide exchange factor 1; MFN1/2: mitofusin 1/2; SIRT4: sirtuin 4; eIF5a: eukaryotic translation initiation factor 5a; FIS1: fission protein 1; MFF: mitochondria fission factor; ATPIF1: ATPase inhibitory factor 1; MTP18: mitochondrial fission process 1,18 kDa; PPARγ: peroxisome proliferator-activated receptor γ; NRF2: nuclear respiratory factors 2.

Mitochondria dynamically regulate their morphology, distribution, and function through fusion and fission. This dynamic equilibrium is crucial for maintaining cellular energy metabolism, quality control, and stress adaptation. Multiple KLFs influence the dynamic balance between fusion and fission by modulating key proteins and adaptor proteins involved in mitochondrial fusion/fission (Figure 4). The regulation of KLF4 on cardiac mitochondrial homeostasis involves many signaling pathways. In addition to the interaction with ERRα and PGC-1α mentioned above to upregulate mitochondrial biogenesis, it is also noticed that the mitochondrial morphology of KLF4-deficient myocardial tissue tends to be more fragmented, enlonged, and heterogeneous accompanied by diminished expression of DRP1, FIS1, MFN2, and OPA1(46-47). In pulmonary artery smooth muscle cells, KLF4 could bind to MFN2 promoter to upregulate gene expression, reverse hypoxia-induced mitochondrial fragmentation, reduce pulmonary vascular remodeling, and exhibit protective impact on pulmonary hypertension (Zhu et al., 2017). In models of liver and myocardial injury (Yu et al., 2020), KLF4 was found to enhance the expression of mitochondrial E3 ligase and membrane-associated ring finger (C3HC4) 5 (March5), which were involved in ubiquitin‐mediated degradation of DRP1, inhibited excessive mitochondrial fission, eventually achieved mitochondrial dynamic balance through the KLF4-MARCH5-DRP1 signaling pathway. In glioblastoma cells, KLF4 could bind to methylated DNA in cis-regulatory regions of the targets genes which induced mitochondrial morphology changes. By KLF4-mCpG binding, neuronal guanine nucleotide exchange factor (NGEF), Rho/Rac guanine nucleotide exchange factor 2 (ARHGEF2), and RAB guanine nucleotide exchange factor 1 (RABGEF1) are upregulated, ultimately promote mitochondrial fusion and further provide a metabolic advantage for glioblastoma cells by improving respiratory reserve capacity under nutritional stress (Wang et al., 2018). Of note, there are some indirect evidences with regard to KLFs in mitochondrial fusion/fission. In epidermoid carcinoma cells A431, Favero et al. found that the localization of KLF4 in the nucleus increased after incubation with deoxynivalenol (DON) for 6 h and followed by a decrease at 24 h. After 24 h of co-incubation, DON induced a concentration-dependent disorder of the mitochondrial network, and mitochondria were almost completely fragmented under concentration of 10 μM. However, it is still unclear whether KLF4 plays a critical role in this process (Del et al., 2021a). In the model of insulin resistance, KLF4 expression was markedly decreased in L6 skeletal muscle cells, and by inhibiting MFN2, it regulated glucose transporter type 4 (GLUT4) expression, which played a role in glucose transport, while the study did not describe its effect on mitochondria (Zhang et al., 2014). In hepatocellular carcinoma cells, Chen et al. proposed that KLF4 promoted SIRT4 transcription by directly binding to the promoter, in turn inhibiting glycolysis and promoting mitochondrial respiration (Chen et al., 2019). More importantly, SIRT4 is localized to the mitochondria and has been confirmed to play a crucial role in various processes, including regulating mitochondrial fusion/fission and mitophagy (Lang et al., 2017; Ding et al., 2022). On the contrary, a report by Wang et al. speculated that KLF4 might be a key to the regulation of mitochondrial morphology mediated by ATPase inhibitory factor 1 (ATPIF1), with the effect of promoting mitochondrial fission and aggravating mitochondrial damage, but further experimental verification is still lacking (Wang K. et al., 2021) (Figure 4).

It has been documented that KLF5 exerts the effect on mitochondrial dynamics. For instance, Ma et al. found that by binding to the promoter, KLF5 activated transcription of the eukaryotic translation initiation factor 5a (eIF5a). Of note, EIF5a plays an important pivotal role in the regulation of mitochondrial dynamics. It directly interacts with MFN1 and significantly upregulates its expression. In addition, it inhibits mitochondrial fission by down-regulating FIS1 and MFF, ultimately contributing to promoting fusion and reducing ROS production as well as inflammatory lesion (Ma et al., 2020).

KLF6 has the protective effect of stabilizing mitochondrial function and inhibiting apoptosis in renal podocytes, and the mechanism may involve the regulation of fusion/fission balance. Podocytes from KLF6-knockout mice showed conversion of mitochondrial morphology from a tubular pattern to an intermediate one. Under doxorubicin stimulation, KLF6-knockout mitochondria also showed more pronounced fragmentation (Mallipattu et al., 2015). Similar mitochondrial morphological alterations following KLF6 deletion were subsequently evident in a diabetic nephropathy model, but the regulatory mechanisms remain poorly understood (Horne et al., 2018).

KLF9 appears to be associated with the reproductive development, cell proliferation and differentiation, and cell cycle regulation (Kang and Lai, 2007). KLF9 is also identified as an important regulator in the redox system, which can be activated by NRF2 to cope with excessive mitochondrial ROS (Zucker et al., 2014). In the central nervous system, KLF9 regulates axonal growth by promoting mitochondrial fission via mitochondrial fission process 1, 18 kDa (MTP18). Knockdown of KLF9 decreased MTP18 expression in retinal ganglion cells, inhibited mitochondrial fission, and increased mitochondrial size (Kreymerman et al., 2019). Other than that, in the latest study by Li et al. (2022), KLF9 was shown to aggravate oxidative stress, inflammatory response and cardiac dysfunction in diabetic cardiomyopathy mice by inhibiting the peroxisome proliferator-activated receptor γ (PPARγ)/NRF2 signaling pathway. The regulatory effect of PPARγ on mitochondrial fusion/fission has been supported by several studies (Zhao et al., 2021; Tol et al., 2016; Zolezzi et al., 2013), indirectly suggesting the involvement of KLF9 in the modulation of mitochondrial dynamic balance.

Megamitochondria was occasionally observed in the subendocardial and interfibrillar positions of KLF15 knockout mice. Different degrees of surface depression might appear on the surface of megamitochondria, indicating that there was mitochondrial fusion-fission imbalance in KLF15 knockout mice, and KLF15 was related to the transcriptional regulation of genes that controlled mitochondrial fission (Tandler et al., 2015).

### 4.3 KLFs in mitophagy

**FIGURE 5:**
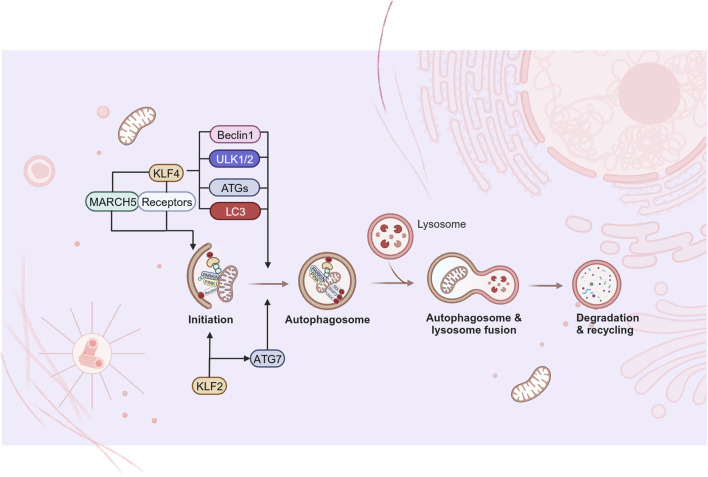
KLFs in mitophagy. Mitophagy refers to the removal of damaged or non-essential mitochondria from cells, which is considered to be one of the organelle‐specific autophagy pathways. Current studies have found that some KLFs are involved in the regulation of the initiation of mitophagy and the formation of autophagosome, while further studies are needed to fill the gaps in other aspects of mitophagy. Figure was created in BioRender. Zhang, M. (2025) https://BioRender.com/b31u319. March5: membrane-associated ring finger (C3HC4) 5; ULK1/2: Unc-51-like kinases 1 and 2; ATG: autophagy-related gene; LC3: microtubule-associated protein light chain 3.

Mitophagy is a selective degradation mechanism for dysfunctional mitochondria. Current research has found that KLF2 and KLF4 can activate a variety of mitophagy-related receptors and autophagy-related proteins, playing a regulatory role in the initiation of mitophagy and the formation of autophagosomes (Figure 5). KLF2 gradually becomes a key “molecular switch” of vascular function due to its regulatory effect on endothelial related genes. In vascular endothelial cells, it seems that that KLF2 and mitophagy are mutually regulated. Coon and colleagues pointed out that laminar shear stress (LSS)-induced mitophagy was a necessary response for high expression of KLF2 (Coon et al., 2022). LSS increased calcium accumulation and ROS production in mitochondria, which further promoted mitochondrial fission and mitophagy. The mitophagy process recruits p62, and p62 binds to MEKK2/3 and MEK5 through its PB1 domain to enhance ERK5 signaling, in turn promoting KLF2 expression. In addition, KLF2 is shown to be positively correlated with level of autophagy and mitophagy during osteoblast differentiation. KLF2 is observed to bind to the promoter of ATG7 and has the positive reciprocal impact on the autophagy-related proteins. Meanwhile, PINK1/PARKIN mediated mitophagy is enhanced, accompanied with decreased mitochondrial ROS and superoxide production (Figure 5).

KLF4 is involved in MQC of cardiomyocytes by regulating mitophagy. Significant mitophagy damage was observed in KLF4 deficient myocardial tissue, however, deletion of KLF4 did not impair mitochondrial aggregation of PINK1/PARKIN and subsequent ubiquitination of mitochondrial proteins or recruitment of p62. It was further revealed that KLF4 could directly transactivate the Unc-51-like kinases 1 and 2 (ULK1/2) promoter and promote phosphorylation/activation of ULK1/2, thereby leading to autophagosome formation (Liao et al., 2015). As previously mentioned, KLF4 upregulated the expression of mitochondrial E3 ligase March5 in models of liver and myocardial injury accompanied by promoting mitophagy (Yu et al., 2020; Li et al., 2020). Mechanisms may involve that protein ubiquitylation by March5 is the initiation of PARKIN recruitment and activation, and it is critical for PINK1/PARKIN-mediated mitophagy (Koyano et al., 2019). Also, KLF4 augments mitophagy by directly up-regulating mitophagy-related receptors. In this regard, Chen et al. (Chen et al., 2008) found a KLF4 binding site on the proximal promoter of BNIP3. In 2020, KLF4 was further confirmed to be essential for BNIP3-mediated mitophagy in mouse embryonic fibroblasts (MEFs) following carbonyl cyanide m-chlorophenylhydrazone (CCCP) stimulation. The study also extended the conclusion to human colorectal cancer cells, confirming the conservation of the positive regulation of BNIP3 expression by KLF4 (Rosencrans et al., 2020). In the field of stem cells research, the reduced mitochondrial number and oxidative phosphorylation of iPSC have been widely recognized (Drews et al., 2012). Xiang et al. proposed that NIX/BNIP3L-mediated mitophagy played a vital role in SKP/SKO (Sox2, KLF4, Pou5f1/Oct4) reprogramming (Xiang et al., 2017). There is also evidence that KLF4 is critically involved in the transcriptional activation of S18-2 in mice and humans, and the mitochondrial ribosomal protein S18-2 located in the mitochondrial matrix could directly bind to PHB2, it located in the IMM and was one of the novel mitophagy receptors independent of PINK1-PARKIN (Mushtaq et al., 2020). Additionally, macro-autophagy is noted to be significantly enhanced during the induction of iPSCs, among which ATG5, ATG7, LC3B, and Beclin1 are identified to be activated by KLF4 (Wu et al., 2015), which provide possible points for the mechanism underlying KLF4 promoting mitophagy.

### 4.4 KLFs in UPRmt

Conclusive evidence elucidating the involvement or regulatory role of KLFs in UPRmt remains lacking. A single published study has focused on the cutaneous toxicity of deoxynivalenol (DON), utilizing proteome/phosphoproteome profiling to demonstrate that DON exposure in epidermal cells triggers marked UPRmt activation. Concurrently, proteasomal proteins, components of the ubiquitination machinery, and critical UPRmt mediators—such as heat shock protein 70 kDa like 1 (HSPA1L) and DnaJ homolog subfamily A member 1 (DNAJA1)—were significantly downregulated, compromising the clearance capacity for mitochondrial unfolded/misfolded proteins. The study also noted reduced KLF4 expression and diminished nuclear localization of KLF4, though the direct mechanistic link to UPRmt dysregulation remains uncharacterized (Del et al., 2021b).

## 5 KLFs-MQC in human diseases

Building upon the established regulatory roles of KLFs in MQC across diverse cells, tissues, and pathophysiological contexts, this section highlights aberrant KLF-mediated MQC modulation in human diseases, further underscoring the therapeutic potential of targeting the KLFs-MQC axis.

### 5.1 Cardiovascular disorders

In both human and rat models of pulmonary arterial hypertension, elevated KLF5 expression correlates with disease severity. Courboulin et al. demonstrated that KLF5 overexpression induces mitochondrial membrane potential hyperpolarization and inhibits apoptosis in pulmonary arterial vascular smooth muscle cells, though the mechanistic involvement of MQC remains unclear (Courboulin et al., 2011). In abdominal aortic aneurysm (AAA), KLF5 downregulation in vascular smooth muscle cells drives vascular senescence. KLF5 deficiency upregulates mitochondrial fission regulators DRP1, FIS1 and mitochondrial fission regulator 1 (Mtfr1) while suppressing MFN1, resulting in pronounced mitochondrial fragmentation. Conversely, inhibiting mitochondrial fission could significantly reduce mtROS production and attenuate AAA progression (Ma et al., 2020). In myocardial fibrosis, mitochondrial dynamic imbalance in cardiac fibroblasts exacerbates pathogenesis. Through loss- and gain-of-function experiments, Zhang et al. identified pathologically elevated KLF6 as a suppressor of MFN1/MFN2 and an inducer of DRP1, driving excessive mitochondrial fission and subsequent cardiac pump failure (Zhang et al., 2024).

### 5.2 Renal pathologies

The KLF-MQC axis is implicated in kidney diseases. In obesity-associated nephropathy, Jin et al. observed reduced renal KLF4 levels correlating with elevated serum creatinine and urea nitrogen. Mechanistically, KLF4 deficiency suppresses PGC1α/PPARγ signaling, impairing mitochondrial biogenesis, respiratory capacity, and antioxidant defenses, thereby exacerbating lipid-induced renal injury (Jin et al., 2020b). In diabetic nephropathy, KLF6 downregulation in glomeruli and podocytes coincides with mitochondrial fragmentation, respiratory dysfunction, and oxidative stress, culminating in podocyte apoptosis (Horne et al., 2018). In chronic kidney disease, KLF4 deficiency in renal macrophages attenuates mitophagy by suppressing PINK1, PARKIN, and BNIP3 expression, promoting pro-inflammatory M1 macrophage infiltration and accelerating renal fibrosis (Cao et al., 2023).

### 5.3 Hepatic and neurological disorders

In liver injury models, KLF4 depletion disrupts mitochondrial dynamics. Yu et al. identified KLF4 as a direct regulator of membrane-associated RING-CH 5 (March5), which ubiquitinates DRP1 to promote its degradation, thereby stabilizing mitochondrial dynamics (Yu et al., 2020). In Parkinson’s disease, KLF4 upregulation impairs autophagic lysosomal degradation, leading to pathogenic mitochondrial accumulation, neuronal apoptosis, and exacerbated behavioral deficits in murine models (Xiao et al., 2024).

### 5.4 Osteoarthritis, cancer and lysosomal storage disorders

In age-related osteoarthritis, chondrocyte-specific KLF10 overexpression represses BNIP3 transcription, disrupting BNIP3-mediated mitophagy and amplifying mitochondrial fragmentation and ROS production, thereby accelerating cartilage degeneration (Shang et al., 2023). In cervical cancer, KLF7 overexpression correlates with poor patient survival, promoting tumor cell proliferation and migration via enhanced mitochondrial abundance (Mao et al., 2024). Mitochondrial dysfunction is a hallmark of lysosomal storage diseases. In models of Niemann-Pick type C (NPC) and acid sphingomyelinase deficiency, KLF2 upregulation suppresses mitochondrial biogenesis by repressing NRF1, NRF2, and TFAM expression, while concurrently impairing respiratory function (Yambire et al., 2019).

These findings collectively emphasize the KLF-MQC axis as a pivotal therapeutic target across diverse diseases, with dysregulated KLFs driving mitochondrial dysfunction through distinct molecular cascades.

## 6 Specific pharmacological modulation of KLFs

It has been document that imbalance of MQC participates in the pathophysiology of various diseases, including Parkinson’s disease (Wang WW. et al., 2021), ischemic heart disease (Xin et al., 2021), stroke (Tian et al., 2022), chronic obstructive pulmonary disease and idiopathic pulmonary fibrosis (Hara et al., 2018), cancer (Vara-Perez et al., 2019), and sepsis-related organ damage (Li et al., 2021; Yu et al., 2022). Given the key regulator of KLFs in MQC, targeted therapy of KLFs may become an emerging treatment for specific pathological states. It has been confirmed that Epigallocatechin-3-gallate, abundant in green tea, promotes mitochondrial fusion and reverses mitochondrial fragmentation through the upregulation of KLF4, resulting in a reduction of hypoxia-induced pulmonary vascular remodeling (Zhu et al., 2017). In recent years, several drugs and compounds have demonstrated the ability to regulate the expression of KLFs, offering new possibilities for improving patient prognosis. However, it remains unsuspected whether MQC plays a critical role in these interventional measures.

As early as 2010, several small molecule inhibitors of KLF5 have been gradually identified using cell-based ultrahigh-throughput screening, such as ML264, SR15006, and SR18662. Among them, ML264 has been confirmed to directly inhibit the activity of KLF5 promoter through luciferase assay. SR15006 and SR18662 have undergone structural modifications in the glycine amide region of ML264. These inhibitors effectively reduce the endogenous KLF5 level of several colorectal cancer cell lines and play a tumor suppressor role (Bialkowska et al., 2010; Kim et al., 2019).

Kenpaullone, a commonly used KLF4 inhibitor in basic study, has been shown to significantly downregulate the mRNA and protein levels of KLF4 in various models, including canine mammary tumor (Tien et al., 2015),non-Hodgkin lymphoma (Montecillo-Aguado et al., 2021) and autoimmune arthritis (Choi et al., 2018). It has been proposed that Kenpaullone can induce the expression of microRNA-182, which targets to BNIP3, a mitophagy receptor to LC3. However, the study did not determine the impact of Kenpaullone on regulating whole MQC system but only show protection from hypoxia-induced mitochondrial fission, excessive ROS generation, and apoptosis (Lee et al., 2016). Likewise, Scott et al. reported that treatment with Kenpaullone increased mitochondrial network area, prevented CCCP-induced exhaustion of mitochondrial membrane potential and excessive mitochondrial fragmentation (Scott et al., 2020). These findings indicate obviously regulation of MQC by Kenpaullone, nonetheless, whether the effect is mediated by KLF4 requires for further illustration.

In summary, although there are limited studies on drug regulation targeting MQC, based on its clear role in KLFs, modulators of KLFs may become potential drugs for regulating mitochondrial function and will be a hot area in future research.

## 7 Discussion

Although most KLFs are ubiquitously expressed in tissues and cells with similar protein structures and conserved zinc finger domains, the expression abundance of each member is different in various tissues. Of note, the differential expression occurs under the regulation of transcriptional and post-transcriptional modifications in different conditions. With the help of the co-regulators combined with amino-terminus, KLFs play a context-dependent role in physiological and pathophysiological processes. MQC appears to be a precise, continuous, and mutually regulated complex network system. Abnormality in any link will destroy the balance and lead to mitochondrial dysfunction, affect energy metabolism, aggravate oxidative stress damage, and bring adverse effects on cell fate.

In this paper, we review the related research findings of KLFs in MQC, but most of the studies are limited to the confirmation and description of the regulatory phenomena. Through extensive literature reading and experiments, we have found that the regulatory role of KLFs in a specific MQC pathway is not constant. The positive and/or negative regulation may depend on cell and tissue specificity as well as environmental stimuli. The finer regulatory mechanisms are still under investigation. In addition, the current study mainly focuses on the regulation of single KLF, whereas in the complex pathophysiological process, whether there is synergy or competition among various KLFs? Similarly, whether the regulatory modes of the KLFs on different MQC links are coordinated or mutually antagonistic? It can be concluded that potential mechanisms concerning the regulation of KLFs in MQC and the functional coordination among KLFs are still a question for future studies. In terms of treatment, drugs that modulate the expression and function of KLFs have been gradually applied to the interventional strategy for tumors, cardiovascular diseases, kidney diseases, and metabolic diseases. However, considering the differences in tissue distribution abundance of KLFs and the diversity, complexity, and tissue specificity of their functions, the development of targeted drugs and the efficacy stability as well as safety of existing drugs in other systems under different pathological environments will be the focus of future evaluation.

In conclusion, KLFs indeed play an important role in the MQC network. In-depth investigation of the structures, regulatory mechanisms, signaling pathways, and targeted strategy for KLFs will be warranted for the transformation from basic research to clinical application.

## References

[B1] Adebayo M. Singh S. Singh A. P. Dasgupta S. (2021). Mitochondrial fusion and fission: the fine-tune balance for cellular homeostasis. Faseb J. 35, e21620. 10.1096/fj.202100067R 34048084 PMC8415099

[B2] Akmeriç E. B. Gerhardt H. (2022). Blood flow meets mitophagy. J. Cell Biol. 221, e202206033. 10.1083/jcb.202206033 35727256 PMC9213090

[B3] Andres A. M. Tucker K. C. Thomas A. Taylor D. J. Sengstock D. Jahania S. M. (2017). Mitophagy and mitochondrial biogenesis in atrial tissue of patients undergoing heart surgery with cardiopulmonary bypass. JCI Insight 2, e89303. 10.1172/jci.insight.89303 28239650 PMC5313075

[B4] Bai P. Cantó C. Oudart H. Brunyánszki A. Cen Y. Thomas C. (2011). PARP-1 inhibition increases mitochondrial metabolism through SIRT1 activation. Cell Metab. 13, 461–468. 10.1016/j.cmet.2011.03.004 21459330 PMC3086520

[B5] Basse A. L. Dixen K. Yadav R. Tygesen M. P. Qvortrup K. Kristiansen K. (2015). Global gene expression profiling of brown to white adipose tissue transformation in sheep reveals novel transcriptional components linked to adipose remodeling. BMC Genomics 16, 215. 10.1186/s12864-015-1405-8 25887780 PMC4407871

[B6] Bialkowska A. Crisp M. Madoux F. Spicer T. Knapinska A. Mercer B. (2010). “ML264: an antitumor agent that potently and selectively inhibits krüppel-like factor five (KLF5) expression: a probe for studying colon cancer development and progression,” in Probe reports from the NIH molecular libraries Program (Bethesda (MD): National Center for Biotechnology Information US). 23762940

[B7] Cao J. Y. Zhang Y. Z. Ding X. F. Sun T. W. (2021). The role of KLF4 in LPS induced cardiomyocyte injury. Chin. J. Emerg. Med. 30, 704–709. 10.3760/cma.j.issn.1671-0282.2021.06.012

[B8] Cao Y. Xiong J. Guan X. Yin S. Chen J. Yuan S. (2023). Paeoniflorin suppresses kidney inflammation by regulating macrophage polarization via KLF4-mediated mitophagy. Phytomedicine 116, 154901. 10.1016/j.phymed.2023.154901 37247587

[B9] Chen X. Ding X. Wu Q. Qi J. Zhu M. Miao C. (2019). Monomethyltransferase SET8 facilitates hepatocellular carcinoma growth by enhancing aerobic glycolysis. Cell Death Dis. 10, 312. 10.1038/s41419-019-1541-1 30952833 PMC6450876

[B10] Chen X. Xu H. Yuan P. Fang F. Huss M. Vega V. B. (2008). Integration of external signaling pathways with the core transcriptional network in embryonic stem cells. Cell 133, 1106–1117. 10.1016/j.cell.2008.04.043 18555785

[B11] Chodari L. Dilsiz Aytemir M. Vahedi P. Alipour M. Vahed S. Z. Khatibi S. M. H. (2021). Targeting mitochondrial biogenesis with polyphenol compounds. Oxid. Med. Cell Longev. 2021, 4946711. 10.1155/2021/4946711 34336094 PMC8289611

[B12] Choi S. Lee K. Jung H. Park N. Kang J. Nam K. H. (2018). Kruppel-like factor 4 positively regulates autoimmune arthritis in mouse models and rheumatoid arthritis in patients via modulating cell survival and inflammation factors of fibroblast-like synoviocyte. Front. Immunol. 9, 1339. 10.3389/fimmu.2018.01339 29997611 PMC6030377

[B13] Choubey V. Zeb A. (2021). Molecular mechanisms and regulation of mammalian mitophagy. Cells 11, 38. 10.3390/cells11010038 35011599 PMC8750762

[B14] Coon B. G. Timalsina S. Astone M. Zhuang Z. W. Fang J. Han J. (2022). A mitochondrial contribution to anti-inflammatory shear stress signaling in vascular endothelial cells. J. Cell Biol. 221, e202109144. 10.1083/jcb.202109144 35695893 PMC9198948

[B15] Courboulin A. Tremblay V. L. Barrier M. Meloche J. Jacob M. H. Chapolard M. (2011). Krüppel-like factor 5 contributes to pulmonary artery smooth muscle proliferation and resistance to apoptosis in human pulmonary arterial hypertension. Respir. Res. 12 (1), 128. 10.1186/1465-9921-12-128 21951574 PMC3193170

[B16] Czakai K. Leonhardt I. Dix A. Bonin M. Linde J. Einsele H. (2016). Krüppel-like Factor 4 modulates interleukin-6 release in human dendritic cells after *in vitro* stimulation with Aspergillus fumigatus and Candida albicans. Sci. Rep. 6, 27990. 10.1038/srep27990 27346433 PMC4921831

[B17] Del F. G. Janker L. Neuditschko B. Hohenbichler J. Kiss E. Woelflingseder L. (2021a). Exploring the dermotoxicity of the mycotoxin deoxynivalenol: combined morphologic and proteomic profiling of human epidermal cells reveals alteration of lipid biosynthesis machinery and membrane structural integrity relevant for skin barrier function. Arch. Toxicol. 95, 2201–2221. 10.1007/s00204-021-03042-y 33890134 PMC8166681

[B18] Del F. G. Janker L. Neuditschko B. Hohenbichler J. Kiss E. Woelflingseder L. (2021b). Exploring the dermotoxicity of the mycotoxin deoxynivalenol: combined morphologic and proteomic profiling of human epidermal cells reveals alteration of lipid biosynthesis machinery and membrane structural integrity relevant for skin barrier function. Arch. Toxicol. 95 (6), 2201–2221. 10.1007/s00204-021-03042-y 33890134 PMC8166681

[B19] Ding L. Li S. Wang F. Xu J. Li S. Wang B. (2021). Berberine improves dietary-induced cardiac remodeling by upregulating Kruppel-like factor 4-dependent mitochondrial function. Biol. Chem. 402, 795–803. 10.1515/hsz-2020-0267 33544461

[B20] Ding Q. Wang Y. Xia S. W. Zhao F. Zhong J. F. Wang H. L. (2022). SIRT4 expression ameliorates the detrimental effect of heat stress via AMPK/mTOR signaling pathway in BMECs. Int. J. Mol. Sci. 23, 13307. 10.3390/ijms232113307 36362094 PMC9658231

[B21] Doddaballapur A. Michalik K. M. Manavski Y. Lucas T. Houtkooper R. H. You X. (2015). Laminar shear stress inhibits endothelial cell metabolism via KLF2-mediated repression of PFKFB3. Arterioscler. Thromb. Vasc. Biol. 35, 137–145. 10.1161/atvbaha.114.304277 25359860

[B22] Dong J. T. (2006). Prevalent mutations in prostate cancer. J. Cell Biochem. 97, 433–447. 10.1002/jcb.20696 16267836

[B23] Drews K. Jozefczuk J. Prigione A. Adjaye J. (2012). Human induced pluripotent stem cells--from mechanisms to clinical applications. J. Mol. Med. Berl. 90, 735–745. 10.1007/s00109-012-0913-0 22643868

[B24] Fan Y. Lu H. Liang W. Hu W. Zhang J. Chen Y. E. (2017). Krüppel-like factors and vascular wall homeostasis. J. Mol. Cell Biol. 9, 352–363. 10.1093/jmcb/mjx037 28992202 PMC5907833

[B25] Gan L. Liu Z. Luo D. Ren Q. Wu H. Li C. (2017). Reduced endoplasmic reticulum stress-mediated autophagy is required for leptin alleviating inflammation in adipose tissue. Front. Immunol. 8, 1507. 10.3389/fimmu.2017.01507 29250056 PMC5715390

[B26] Ghaleb A. M. Yang V. W. (2017). Krüppel-like factor 4 (KLF4): what we currently know. Gene 611, 27–37. 10.1016/j.gene.2017.02.025 28237823 PMC5391259

[B27] Guan W. K. Lv J. Yang C. X. (2021). A new pathway for mitochondrial quality control: mitochondrial-derived vesicle. Acta Anat. Sin. 52, 152–156. 10.16098/j.issn.0529-1356.2021.01.025

[B28] Hara H. Kuwano K. Araya J. (2018). Mitochondrial quality control in COPD and IPF. Cells 7, 86. 10.3390/cells7080086 30042371 PMC6115906

[B29] Horne S. J. Vasquez J. M. Guo Y. Ly V. Piret S. E. Leonardo A. R. (2018). Podocyte-specific loss of krüppel-like factor 6 increases mitochondrial injury in diabetic kidney disease. Diabetes 67, 2420–2433. 10.2337/db17-0958 30115650 PMC6198342

[B30] Hsieh P. N. Fan L. Sweet D. R. Jain M. K. (2019). The krüppel-like factors and control of energy homeostasis. Endocr. Rev. 40, 137–152. 10.1210/er.2018-00151 30307551 PMC6334632

[B31] Jiao H. Jiang D. Hu X. Du W. Ji L. Yang Y. (2021). Mitocytosis, a migrasome-mediated mitochondrial quality-control process. Cell 184, 2896–2910.e13. 10.1016/j.cell.2021.04.027 34048705

[B32] Jin L. Ye H. Pan M. Chen Y. Ye B. Zheng Y. (2020a). Kruppel-like factor 4 improves obesity-related nephropathy through increasing mitochondrial biogenesis and activities. J. Cell Mol. Med. 24, 1200–1207. 10.1111/jcmm.14628 31800161 PMC6991690

[B33] Jin L. Ye H. Pan M. Chen Y. Ye B. Zheng Y. (2020b). Kruppel-like factor 4 improves obesity-related nephropathy through increasing mitochondrial biogenesis and activities. J. Cell Mol. Med. 24 (2), 1200–1207. 10.1111/jcmm.14628 31800161 PMC6991690

[B34] Kammoun M. Piquereau J. Nadal-Desbarats L. Même S. Beuvin M. Bonne G. (2020). Novel role of Tieg1 in muscle metabolism and mitochondrial oxidative capacities. Acta Physiol. (Oxf) 228, e13394. 10.1111/apha.13394 31560161

[B35] Kang L. Lai M. D. (2007). BTEB/KLF9 and its transcriptional regulation. Yi Chuan 29, 515–522. 10.1360/yc-007-0515 17548317

[B36] Kapoor N. Niu J. Saad Y. Kumar S. Sirakova T. Becerra E. (2015). Transcription factors STAT6 and KLF4 implement macrophage polarization via the dual catalytic powers of MCPIP. J. Immunol. 194, 6011–6023. 10.4049/jimmunol.1402797 25934862 PMC4458412

[B37] Kim J. Wang C. de Sabando A. R. Cole H. L. Huang T. J. Yang J. (2019). The novel small-molecule SR18662 efficiently inhibits the growth of colorectal cancer *in vitro* and *in vivo* . Mol. Cancer Ther. 18, 1973–1984. 10.1158/1535-7163.Mct-18-1366 31358661 PMC6825545

[B38] Kopp J. B. (2015). Loss of Krüppel-like factor 6 cripples podocyte mitochondrial function. J. Clin. Invest 125, 968–971. 10.1172/jci80280 25689255 PMC4362253

[B39] Koritschoner N. P. Bocco J. L. Panzetta-Dutari G. M. Dumur C. I. Flury A. Patrito L. C. (1997). A novel human zinc finger protein that interacts with the core promoter element of a TATA box-less gene. J. Biol. Chem. 272, 9573–9580. 10.1074/jbc.272.14.9573 9083102

[B40] Koyano F. Yamano K. Kosako H. Tanaka K. Matsuda N. (2019). Parkin recruitment to impaired mitochondria for nonselective ubiquitylation is facilitated by MITOL. J. Biol. Chem. 294, 10300–10314. 10.1074/jbc.RA118.006302 31110043 PMC6664184

[B41] Kreymerman A. Buickians D. N. Nahmou M. M. Tran T. Galvao J. Wang Y. (2019). MTP18 is a novel regulator of mitochondrial fission in CNS neuron development, axonal growth, and injury responses. Sci. Rep. 9, 10669. 10.1038/s41598-019-46956-5 31337818 PMC6650498

[B42] Kyriazis I. D. Hoffman M. Gaignebet L. Lucchese A. M. Markopoulou E. Palioura D. (2021). KLF5 is induced by FOXO1 and causes oxidative stress and diabetic cardiomyopathy. Circ. Res. 128, 335–357. 10.1161/circresaha.120.316738 33539225 PMC7870005

[B43] Lang A. Anand R. Altinoluk-Hambüchen S. Ezzahoini H. Stefanski A. Iram A. (2017). SIRT4 interacts with OPA1 and regulates mitochondrial quality control and mitophagy. Aging (Albany NY) 9, 2163–2189. 10.18632/aging.101307 29081403 PMC5680561

[B44] Lee S. Y. Lee S. Choi E. Ham O. Lee C. Y. Lee J. (2016). Small molecule-mediated up-regulation of microRNA targeting a key cell death modulator BNIP3 improves cardiac function following ischemic injury. Sci. Rep. 6, 23472. 10.1038/srep23472 27008992 PMC4806297

[B45] Leites E. P. Morais V. A. (2018). Mitochondrial quality control pathways: PINK1 acts as a gatekeeper. Biochem. Biophys. Res. Commun. 500, 45–50. 10.1016/j.bbrc.2017.06.096 28647367

[B46] Li C. Wang W. Xie S. S. Ma W. X. Fan Q. W. Chen Y. (2021). The programmed cell death of macrophages, endothelial cells, and tubular epithelial cells in sepsis-AKI. Front. Med. (Lausanne) 8, 796724. 10.3389/fmed.2021.796724 34926535 PMC8674574

[B47] Li F. Peng J. Feng H. Yang Y. Gao J. Liu C. (2022). KLF9 aggravates streptozotocin-induced diabetic cardiomyopathy by inhibiting pparγ/NRF2 signalling. Cells 11, 3393. 10.3390/cells11213393 36359788 PMC9656075

[B48] Li H. X. Han M. Bernier M. Zheng B. Sun S. G. Su M. (2010). Krüppel-like factor 4 promotes differentiation by transforming growth factor-beta receptor-mediated Smad and p38 MAPK signaling in vascular smooth muscle cells. J. Biol. Chem. 285, 17846–17856. 10.1074/jbc.M109.076992 20375011 PMC2878548

[B49] Li Q. Yu Z. Xiao D. Wang Y. Zhao L. An Y. (2020). Baicalein inhibits mitochondrial apoptosis induced by oxidative stress in cardiomyocytes by stabilizing MARCH5 expression. J. Cell Mol. Med. 24, 2040–2051. 10.1111/jcmm.14903 31880404 PMC6991701

[B50] Li X. Liu X. Xu Y. Liu J. Xie M. Ni W. (2014). KLF5 promotes hypoxia-induced survival and inhibits apoptosis in non-small cell lung cancer cells via HIF-1α. Int. J. Oncol. 45, 1507–1514. 10.3892/ijo.2014.2544 25051115

[B51] Li Z. Jia Y. Han S. Wang X. Han F. Zhang J. (2018). Klf4 alleviates lipopolysaccharide-induced inflammation by inducing expression of MCP-1 induced protein 1 to deubiquitinate TRAF6. Cell Physiol. Biochem. 47, 2278–2290. 10.1159/000491538 29975947

[B52] Liang L. Huang Y. Mo Z. Q. Zhu H. Ma Y. L. (2018). Expression and clinical significance of KLF gene family in breast cancer. Genomics Appl. Biol. 37, 2257–2265. 10.13417/j.gab.037.002257

[B53] Liao X. Zhang R. Lu Y. Prosdocimo D. A. Sangwung P. Zhang L. (2015). Kruppel-like factor 4 is critical for transcriptional control of cardiac mitochondrial homeostasis. J. Clin. Invest 125, 3461–3476. 10.1172/jci79964 26241060 PMC4588311

[B54] Liu J. Liu Y. Zhang H. Chen G. Wang K. Xiao X. (2008). KLF4 promotes the expression, translocation, and releas eof HMGB1 in RAW264.7 macrophages in response to LPS. Shock 30, 260–266. 10.1097/shk.0b013e318162bef7 18197146

[B55] Liu J. Yang T. Liu Y. Zhang H. Wang K. Liu M. (2012). Krüppel-like factor 4 inhibits the expression of interleukin-1 beta in lipopolysaccharide-induced RAW264.7 macrophages. FEBS Lett. 586, 834–840. 10.1016/j.febslet.2012.02.003 22449968

[B56] Liu J. Zhang H. Liu Y. Wang K. Feng Y. Liu M. (2007). KLF4 regulates the expression of interleukin-10 in RAW264.7 macrophages. Biochem. Biophys. Res. Commun. 362, 575–581. 10.1016/j.bbrc.2007.07.157 17719562

[B57] Lopez-Ramirez M. A. McCurdy S. Li W. Haynes M. K. Hale P. Francisco K. (2021). Inhibition of the HEG1-KRIT1 interaction increases KLF4 and KLF2 expression in endothelial cells. FASEB Bioadv 3, 334–355. 10.1096/fba.2020-00141 33977234 PMC8103725

[B58] Loss O. Stephenson F. A. (2017). Developmental changes in trak-mediated mitochondrial transport in neurons. Mol. Cell Neurosci. 80, 134–147. 10.1016/j.mcn.2017.03.006 28300646 PMC5400476

[B59] Ma D. Zheng B. Liu H. L. Zhao Y. B. Liu X. Zhang X. H. (2020). Klf5 down-regulation induces vascular senescence through eIF5a depletion and mitochondrial fission. PLoS Biol. 18, e3000808. 10.1371/journal.pbio.3000808 32817651 PMC7462304

[B60] Mallipattu S. K. Horne S. J. D'Agati V. Narla G. Liu R. Frohman M. A. (2015). Krüppel-like factor 6 regulates mitochondrial function in the kidney. J. Clin. Invest 125, 1347–1361. 10.1172/jci77084 25689250 PMC4362257

[B61] Mao Y. Li H. Xu G. Tian J. Chen Y. Zhang Z. (2024). Alpha-lipoic acid targets KLF7 expression to inhibit cervical cancer progression. Acta Biochim. Biophys. Sin. (Shanghai) 57 (2), 237–249. 17. 10.3724/abbs.2024212 39696984 PMC11877147

[B62] Mas C. Lussier-Price M. Soni S. Morse T. Arseneault G. Di Lello P. (2011). Structural and functional characterization of an atypical activation domain in erythroid Kruppel-like factor (EKLF). Proc. Natl. Acad. Sci. U. S. A. 108, 10484–10489. 10.1073/pnas.1017029108 21670263 PMC3127900

[B63] Memon A. Lee W. K. (2018). KLF10 as a tumor suppressor gene and its TGF-β signaling. Cancers (Basel) 10, 161. 10.3390/cancers10060161 29799499 PMC6025274

[B64] Montecillo-Aguado M. Morales-Martínez M. Huerta-Yepez S. Vega M. I. (2021). KLF4 inhibition by Kenpaullone induces cytotoxicity and chemo sensitization in B-NHL cell lines via YY1 independent. Leuk. Lymphoma 62, 1422–1431. 10.1080/10428194.2020.1869960 33410342

[B65] Mushtaq M. Kovalevska L. Darekar S. Abramsson A. Zetterberg H. Kashuba V. (2020). Cell stemness is maintained upon concurrent expression of RB and the mitochondrial ribosomal protein S18-2. Proc. Natl. Acad. Sci. U. S. A. 117, 15673–15683. 10.1073/pnas.1922535117 32571933 PMC7355020

[B66] Nishimura K. Aizawa S. Nugroho F. L. Shiomitsu E. Tran Y. T. H. Bui P. L. (2017). A role for KLF4 in promoting the metabolic shift via TCL1 during induced pluripotent stem cell generation. Stem Cell Rep. 8 (8), 787–801. 10.1016/j.stemcr.2017.01.026 PMC5355680 28262547

[B67] Oishi Y. Manabe I. (2018). Krüppel-like factors in metabolic homeostasis and cardiometabolic disease. Front. Cardiovasc Med. 5, 69. 10.3389/fcvm.2018.00069 29942807 PMC6004387

[B68] Onishi M. Yamano K. Sato M. Matsuda N. Okamoto K. (2021). Molecular mechanisms and physiological functions of mitophagy. Embo J. 40, e104705. 10.15252/embj.2020104705 33438778 PMC7849173

[B69] Palioura D. Lazou A. Drosatos K. (2022). Krüppel-like factor (KLF)5: an emerging foe of cardiovascular health. J. Mol. Cell Cardiol. 163, 56–66. 10.1016/j.yjmcc.2021.10.002 34653523 PMC8816822

[B70] Pei J. Grishin N. V. (2013). A new family of predicted Krüppel-like factor genes and pseudogenes in placental mammals. PLoS One 8, e81109. 10.1371/journal.pone.0081109 24244731 PMC3820594

[B71] Perkins A. Xu X. Higgs D. R. Patrinos G. P. Arnaud L. Bieker J. J. (2016). Krüppeling erythropoiesis: an unexpected broad spectrum of human red blood cell disorders due to KLF1 variants. Blood 127, 1856–1862. 10.1182/blood-2016-01-694331 26903544 PMC4832505

[B72] Picca A. Guerra F. Calvani R. Coelho-Junior H. J. Bossola M. Landi F. (2020). Generation and release of mitochondrial-derived vesicles in health, aging and disease. J. Clin. Med. 9, 1440. 10.3390/jcm9051440 32408624 PMC7290979

[B73] Popov L. D. (2020). Mitochondrial biogenesis: an update. J. Cell Mol. Med. 24, 4892–4899. 10.1111/jcmm.15194 32279443 PMC7205802

[B74] Romanello V. (2020). The interplay between mitochondrial morphology and myomitokines in aging sarcopenia. Int. J. Mol. Sci. 22 (1), 91. 10.3390/ijms22010091 33374852 PMC7796142

[B75] Rosencrans W. M. Walsh Z. H. Houerbi N. Blum A. Belew M. Liu C. (2020). Cells deficient for Krüppel-like factor 4 exhibit mitochondrial dysfunction and impaired mitophagy. Eur. J. Cell Biol. 99, 151061. 10.1016/j.ejcb.2019.151061 31839365

[B76] Scott H. L. Buckner N. Fernandez-Albert F. Pedone E. Postiglione L. Shi G. (2020). A dual druggable genome-wide siRNA and compound library screening approach identifies modulators of parkin recruitment to mitochondria. J. Biol. Chem. 295, 3285–3300. 10.1074/jbc.RA119.009699 31911436 PMC7062187

[B77] Shang J. Lin N. Peng R. Jiang N. Wu B. Xing B. (2023). Inhibition of Klf10 attenuates oxidative stress-induced senescence of chondrocytes via modulating mitophagy. Molecules 28 (3), 924. 10.3390/molecules28030924 36770589 PMC9921806

[B78] Shi J. Yu J. Zhang Y. Wu L. Dong S. Wu L. (2019). PI3K/Akt pathway-mediated HO-1 induction regulates mitochondrial quality control and attenuates endotoxin-induced acute lung injury. Lab. Invest 99, 1795–1809. 10.1038/s41374-019-0286-x 31570770

[B79] Shields J. M. Christy R. J. Yang V. W. (1996). Identification and characterization of a gene encoding a gut-enriched Krüppel-like factor expressed during growth arrest. J. Biol. Chem. 271, 20009–20017. 10.1074/jbc.271.33.20009 8702718 PMC2330254

[B80] Steketee M. B. Moysidis S. N. Weinstein J. E. Kreymerman A. Silva J. P. Iqbal S. (2012). Mitochondrial dynamics regulate growth cone motility, guidance, and neurite growth rate in perinatal retinal ganglion cells *in vitro* . Invest Ophthalmol. Vis. Sci. 53, 7402–7411. 10.1167/iovs.12-10298 23049086 PMC3484733

[B81] Syafruddin S. E. Mohtar M. A. Wan M. Nazarie W. F. Low T. Y. (2020). Two sides of the same coin: the roles of KLF6 in physiology and pathophysiology. Biomolecules 10, 1378. 10.3390/biom10101378 32998281 PMC7601070

[B82] Takashima Y. Guo G. Loos R. Nichols J. Ficz G. Krueger F. (2014). Resetting transcription factor control circuitry toward ground-state pluripotency in human. Cell 158, 1254–1269. 10.1016/j.cell.2014.08.029 25215486 PMC4162745

[B83] Tandler B. Fujioka H. Hoppel C. L. Haldar S. M. Jain M. K. (2015). Megamitochondria in cardiomyocytes of a knockout (Klf15-/-) mouse. Ultrastruct. Pathol. 39, 336–339. 10.3109/01913123.2015.1042610 26111268 PMC4827860

[B84] Tavares C. D. J. Aigner S. Sharabi K. Sathe S. Mutlu B. Yeo G. W. (2020). Transcriptome-wide analysis of PGC-1α-binding RNAs identifies genes linked to glucagon metabolic action. Proc. Natl. Acad. Sci. U. S. A. 117, 22204–22213. 10.1073/pnas.2000643117 32848060 PMC7486754

[B85] Tedesco L. Rossi F. Ragni M. Ruocco C. Brunetti D. Carruba M. O. (2020). A special amino-acid formula tailored to boosting cell respiration prevents mitochondrial dysfunction and oxidative stress caused by doxorubicin in mouse cardiomyocytes. Nutrients 12, 282. 10.3390/nu12020282 31973180 PMC7071384

[B86] Tian H. Chen X. Liao J. Yang T. Cheng S. Mei Z. (2022). Mitochondrial quality control in stroke: from the mechanisms to therapeutic potentials. J. Cell Mol. Med. 26, 1000–1012. 10.1111/jcmm.17189 35040556 PMC8831937

[B87] Tien Y. T. Chang M. H. Chu P. Y. Lin C. S. Liu C. H. Liao A. T. (2015). Downregulation of the KLF4 transcription factor inhibits the proliferation and migration of canine mammary tumor cells. Vet. J. 205, 244–253. 10.1016/j.tvjl.2014.12.031 25616642

[B88] Tol M. J. Ottenhoff R. van Eijk M. Zelcer N. Aten J. Houten S. M. (2016). A pparγ-bnip3 Axis couples adipose mitochondrial fusion-fission balance to systemic insulin sensitivity. Diabetes 65, 2591–2605. 10.2337/db16-0243 27325287 PMC5001173

[B89] Tong L. Tang C. Cai C. Guan X. (2020). Upregulation of the microRNA rno-miR-146b-5p may be involved in the development of intestinal injury through inhibition of Kruppel-like factor 4 in intestinal sepsis. Bioengineered 11, 1334–1349. 10.1080/21655979.2020.1851476 33200654 PMC8291882

[B90] Torres A. K. Fleischhart V. Inestrosa N. C. (2024). Mitochondrial unfolded protein response (UPRmt): what we know thus far. Front. Cell Dev. Biol. 12, 1405393. 10.3389/fcell.2024.1405393 38882057 PMC11176431

[B91] Tsoyi K. Geldart A. M. Christou H. Liu X. Chung S. W. Perrella M. A. (2015). Elk-3 is a KLF4-regulated gene that modulates the phagocytosis of bacteria by macrophages. J. Leukoc. Biol. 97, 171–180. 10.1189/jlb.4A0214-087R 25351511 PMC4377825

[B92] Vara-Perez M. Felipe-Abrio B. Agostinis P. (2019). Mitophagy in cancer: a tale of adaptation. Cells 8, 493. 10.3390/cells8050493 31121959 PMC6562743

[B93] Wang J. Zhou H. (2020). Mitochondrial quality control mechanisms as molecular targets in cardiac ischemia-reperfusion injury. Acta Pharm. Sin. B 10, 1866–1879. 10.1016/j.apsb.2020.03.004 33163341 PMC7606115

[B94] Wang K. Chen H. Zhou Z. Zhang H. Zhou H. J. Min W. (2021a). ATPIF1 maintains normal mitochondrial structure which is impaired by CCM3 deficiency in endothelial cells. Cell Biosci. 11, 11. 10.1186/s13578-020-00514-z 33422124 PMC7796565

[B95] Wang S. Shi X. Wei S. Ma D. Oyinlade O. Lv S. Q. (2018). Krüppel-like factor 4 (KLF4) induces mitochondrial fusion and increases spare respiratory capacity of human glioblastoma cells. J. Biol. Chem. 293, 6544–6555. 10.1074/jbc.RA117.001323 29507094 PMC5925822

[B96] Wang W. W. Han R. He H. J. Li J. Chen S. Y. Gu Y. (2021b). Administration of quercetin improves mitochondria quality control and protects the neurons in 6-OHDA-lesioned Parkinson's disease models. Aging (Albany NY) 13, 11738–11751. 10.18632/aging.202868 33878030 PMC8109056

[B97] Wani M. A. Wert S. E. Lingrel J. B. (1999). Lung Kruppel-like factor, a zinc finger transcription factor, is essential for normal lung development. J. Biol. Chem. 274, 21180–21185. 10.1074/jbc.274.30.21180 10409672

[B98] Wara A. K. Wang S. Wu C. Fang F. Haemmig S. Weber B. N. (2020). KLF10 deficiency in CD4(+) T cells triggers obesity, insulin resistance, and fatty liver. Cell Rep. 33, 108550. 10.1016/j.celrep.2020.108550 33378664 PMC7816773

[B99] Wu Y. Li Y. Zhang H. Huang Y. Zhao P. Tang Y. (2015). Autophagy and mTORC1 regulate the stochastic phase of somatic cell reprogramming. Nat. Cell Biol. 17, 715–725. 10.1038/ncb3172 25985393

[B100] Xiang G. Yang L. Long Q. Chen K. Tang H. Wu Y. (2017). BNIP3L-dependent mitophagy accounts for mitochondrial clearance during 3 factors-induced somatic cell reprogramming. Autophagy 13, 1543–1555. 10.1080/15548627.2017.1338545 28722510 PMC5612220

[B101] Xiao X. Tang T. Bi M. Liu J. Liu M. Jiao Q. (2024). GHSR deficiency exacerbates Parkinson's disease pathology by impairing autophagy. Redox Biol. 76, 103322. 10.1016/j.redox.2024.103322 39180981 PMC11388265

[B102] Xin Y. Zhang X. Li J. Gao H. Li J. Li J. (2021). New Insights into the role of mitochondria quality control in ischemic heart disease. Front. Cardiovasc Med. 8, 774619. 10.3389/fcvm.2021.774619 34901234 PMC8661033

[B103] Yambire K. F. Fernandez-Mosquera L. Steinfeld R. Mühle C. Ikonen E. Milosevic I. (2019). Mitochondrial biogenesis is transcriptionally repressed in lysosomal lipid storage diseases. Elife 8, e39598. 10.7554/eLife.39598 30775969 PMC6379092

[B104] Yan J. Wang A. Cao J. Chen L. (2020). Apelin/APJ system: an emerging therapeutic target for respiratory diseases. Cell Mol. Life Sci. 77, 2919–2930. 10.1007/s00018-020-03461-7 32128601 PMC11105096

[B105] Yang C. Xiao X. Huang L. Zhou F. Chen L. H. Zhao Y. Y. (2021). Role of Kruppel-like factor 4 in atherosclerosis. Clin. Chim. Acta 512, 135–141. 10.1016/j.cca.2020.11.002 33181148

[B106] Yeo J. C. Jiang J. Tan Z. Y. Yim G. R. Ng J. H. Göke J. (2014). Klf2 is an essential factor that sustains ground state pluripotency. Cell Stem Cell 14, 864–872. 10.1016/j.stem.2014.04.015 24905170

[B107] Yin X. M. Ding W. X. (2013). The reciprocal roles of PARK2 and mitofusins in mitophagy and mitochondrial spheroid formation. Autophagy 9, 1687–1692. 10.4161/auto.24871 24162069

[B108] Yu Z. Li Q. Wang Y. Li P. (2020). A potent protective effect of baicalein on liver injury by regulating mitochondria-related apoptosis. Apoptosis 25, 412–425. 10.1007/s10495-020-01608-2 32409930

[B109] Yu Z. Xiao Z. Guan L. Bao P. Yu Y. Liang Y. (2022). Translocation of gasdermin D induced mitochondrial injury and mitophagy mediated quality control in lipopolysaccharide related cardiomyocyte injury. Clin. Transl. Med. 12, e1002. 10.1002/ctm2.1002 36030524 PMC9420421

[B110] Zhang R. Shen Y. Zhou L. Sangwung P. Fujioka H. Zhang L. (2017). Short-term administration of Nicotinamide Mononucleotide preserves cardiac mitochondrial homeostasis and prevents heart failure. J. Mol. Cell Cardiol. 112, 64–73. 10.1016/j.yjmcc.2017.09.001 28882480 PMC6257991

[B111] Zhang T. Ge H. Song Q. Yang G. Li A. (2024). KLF6 aggravates myocardial fibrosis by promoting mitochondrial division. Pak J. Pharm. Sci. 37 (3), 669–679. 39340858

[B112] Zhang Z. Zhao Z. Y. Wang C. (2014). KLF4 ameliorates Palmitate-induced insulin resistance partially by up-regulating Mfn2 expression in L6 skeleton muscle cells. Chin. J. Gerontol. 21, 6076–6078. 10.3969/j.issn.1005-9202.2014.21.064

[B113] Zhao C. C. Xu J. Xie Q. M. Zhang H. Y. Fei G. H. Wu H. M. (2021). Abscisic acid suppresses the activation of NLRP3 inflammasome and oxidative stress in murine allergic airway inflammation. Phytother. Res. 35, 3298–3309. 10.1002/ptr.7051 33570219

[B114] Zhao L. Zhang Q. Liang J. Li J. Tan X. Tang N. (2019). Astrocyte elevated gene-1 induces autophagy in diabetic cardiomyopathy through upregulation of KLF4. J. Cell Biochem. 120, 9709–9715. 10.1002/jcb.28249 30520133

[B115] Zhou L. Li Q. Chen A. Liu N. Chen N. Chen X. (2019). KLF15-activating Twist2 ameliorated hepatic steatosis by inhibiting inflammation and improving mitochondrial dysfunction via NF-κB-FGF21 or SREBP1c-FGF21 pathway. Faseb J. 33, 14254–14269. 10.1096/fj.201901347RR 31648561

[B116] Zhu T. T. Zhang W. F. Luo P. He F. Ge X. Y. Zhang Z. (2017). Epigallocatechin-3-gallate ameliorates hypoxia-induced pulmonary vascular remodeling by promoting mitofusin-2-mediated mitochondrial fusion. Eur. J. Pharmacol. 809, 42–51. 10.1016/j.ejphar.2017.05.003 28478070

[B117] Zolezzi J. M. Silva-Alvarez C. Ordenes D. Godoy J. A. Carvajal F. J. Santos M. J. (2013). Peroxisome proliferator-activated receptor (PPAR) γ and PPARα agonists modulate mitochondrial fusion-fission dynamics: relevance to reactive oxygen species (ROS)-related neurodegenerative disorders? PLoS One 8, e64019. 10.1371/journal.pone.0064019 23675519 PMC3652852

[B118] Zucker S. N. Fink E. E. Bagati A. Mannava S. Bianchi-Smiraglia A. Bogner P. N. (2014). Nrf2 amplifies oxidative stress via induction of Klf9. Mol. Cell 53, 916–928. 10.1016/j.molcel.2014.01.033 24613345 PMC4049522

